# Trends in HDL Cholesterol and Their Association with Dietary Carbohydrate Reduction Among Korean Adults: A Serial Cross-Sectional Trend Analysis Using KNHANES 2014–2023

**DOI:** 10.3390/nu18071045

**Published:** 2026-03-25

**Authors:** Myung-Gwan Kim, Hyun Wook Han

**Affiliations:** 1 Department of Biomedical Informatics, Graduate School of Medicine, CHA University, Seongnam 13488, Republic of Korea; curein@naver.com; 2 Institute for Biomedical Informatics, Graduate School of Medicine, CHA University, Seongnam 13488, Republic of Korea

**Keywords:** HDL cholesterol, dietary habits, lifestyle, risk factors, public health

## Abstract

**Purpose:** Low HDL cholesterol (HDL-C) is a common lipid abnormality among East Asian populations, including Koreans, and is closely associated with increased cardiovascular disease risk. This has traditionally been linked to high-carbohydrate dietary patterns. Therefore, it is essential to assess how recent changes in dietary and lifestyle habits in Korea have influenced HDL-C levels. **Methods:** This study utilized data from the Korea National Health and Nutrition Examination Survey (KNHANES), conducted from 2014 to 2023. Using a complex sampling method, we analyzed annual trends in carbohydrate intake and HDL-C levels in Korean adults (aged 20–59 years), stratified by gender (male and female) and age (20–39 and 40–59 years), and identified determinants such as demographic factors, health behaviors, and dietary intake. **Results:** The primary factor in the increase in HDL-C levels was a reduction in carbohydrate intake. When analyzed by age and gender, this significant upward trend in HDL-C levels was consistently observed across all four groups: young men, middle-aged men, young women, and middle-aged women. However, the increase in obesity, indicated by increased BMI and waist circumference, had a negative impact on the improvement in HDL-C levels. The recent improvement in HDL-C levels in Korean adults can be attributed to the successful national dietary policy (reducing carbohydrate intake and increasing healthy fat intake). **Conclusions:** Public health policies should thus continue emphasizing healthy dietary practices, strengthening smoking cessation initiatives, and managing obesity. These findings suggest similar dietary and lifestyle interventions could effectively reduce cardiovascular disease risk in other Asian populations undergoing comparable dietary transitions.

## 1. Introduction

Historically, high-density lipoprotein cholesterol (HDL-C) has been colloquially termed ‘good cholesterol’ due to its inverse epidemiological associations with atherosclerotic cardiovascular disease (CVD) [1]. However, the causal role of HDL-C mass in directly preventing CVD has been heavily scrutinized. While HDL particles participate in reverse cholesterol transport and exhibit anti-inflammatory and antioxidant properties in vitro, the direct translation of these functions to clinical cardiovascular protection remains complex. Epidemiologically, rather than being a direct causal determinant, low HDL-C is now primarily recognized as a key component of metabolic syndrome and a powerful biomarker for poor cardiometabolic health [1,2]. However, recent large-scale randomized controlled trials and Mendelian randomization studies have prompted a paradigm shift, demonstrating that pharmacologically raising HDL-C levels does not directly translate to improved cardiovascular outcomes [3]. Consequently, contemporary evidence highlights that the quality, composition, and functionality of HDL particles—assessed via proteomic and lipidomic profiling—are more critical determinants of cardiovascular protection than HDL-C quantity alone [4]. Nevertheless, at the population level, low HDL-C mass remains a robust and highly accessible proxy marker for adverse metabolic phenotypes, insulin resistance, and poor lifestyle habits.

In East Asian populations, including South Korea, a phenotype of low HDL-C has traditionally been prevalent, largely attributed to diets rich in refined carbohydrates and low in healthy fats [5]. However, South Korea has recently undergone rapid lifestyle and nutritional transitions. Notably, smoking rates have declined, and national health campaigns have successfully promoted weight management and shifting dietary patterns toward reduced refined carbohydrates and increased protein and unsaturated fats [6,7,8,9,10,11]. These changes would be expected to raise HDL-C levels at the population level, reflecting broader metabolic improvements, since higher unsaturated fat intake and reduced refined carbohydrates can favorably increase HDL-C [12,13]. On the other hand, rising trends in obesity and physical inactivity—potentially exacerbated by recent societal changes—could negatively impact HDL [14]. Understanding how HDL-C levels have evolved in the Korean population during this period of transition is important for evaluating the net impact of health policies and lifestyle shifts and for guiding future interventions.

In this study, we analyzed national big data from 2014 to 2023 to examine trends in HDL-C levels among Korean adults and to investigate the determinants of HDL-C in this population. We hypothesized that HDL-C has increased over time and that this increase is associated with improvements in diet (lower carbohydrate intake) and lifestyle (less smoking), outweighing adverse factors like weight gain. We also sought to quantify the contributions of various factors—such as demographic characteristics, diet, and physical activity—to HDL-C variation. By framing these findings in a public health context, we aim to explore how tracking HDL-C as a population-level indicator can inform strategies for metabolic health management and cardiovascular risk reduction in Korea and similar settings.

## 2. Methods

### Study Design and Data Source

We conducted a trend analysis and cross-sectional determinant study using data from the Korea National Health and Nutrition Examination Survey (KNHANES) [15] for the years 2014–2023. KNHANES is an ongoing nationwide surveillance program that uses a stratified, multistage probability sampling design to select a representative sample of the non-institutionalized Korean population each year [16,17]. The survey consists of three components: a health interview, a health examination, and a nutrition survey. For this analysis, we pooled 10 consecutive annual surveys to assess temporal trends. Each year’s survey employs new sample households drawn from census units, allowing cross-sectional estimates for that year; combining years enabled us to increase statistical power for determinants [16,17].

## 3. Population and Variables

### 3.1. Study Population

This study analyzed data from the KNHANES 2014–2023. From the total participants, we excluded individuals aged under 20 or over 60 years, pregnant women, and those with missing data on lipid profiles, anthropometric measurements, or key covariates. Consequently, a total of 28,101 adults were included in the final analysis. To address the distinct physiological and lifestyle characteristics associated with gender and life stages, as requested by rigorous methodological standards, we stratified the study population into four subgroups: men aged 20–39 (*n* = 5016), women aged 20–39 (*n* = 6434), men aged 40–59 (*n* = 6693), and women aged 40–59 (*n* = 9958). Detailed demographic and physical characteristics of the participants are presented in Section 5.

### 3.2. Criteria of Inclusion and Exclusion

The inclusion criteria for this study were participants aged 20 to 59 years who participated in the KNHANES 2014–2023 survey. The exclusion criteria were as follows:

(1) Individuals aged under 20 years or over 60 years.
(2) Individuals with missing data on the dependent variable (HDL-C).
(3) Individuals with missing data on major covariates.

Consequently, out of the initial 74,952 participants, 46,851 were excluded, leaving 28,101 participants for the final analysis.

### 3.3. Variable Selection

The primary outcome of interest was serum HDL cholesterol, measured enzymatically after an 8–12 h fast. Consistent measurement techniques and quality control were applied over the years by the central laboratory according to standardized protocols. Key exposure variables for determinant analysis were chosen based on known or hypothesized influences on HDL-C. Demographic factors included sex, age, education level, and household income. Lifestyle factors included smoking status (current smoker vs. non-smoker), alcohol drinking status (yes vs. no, defined as regular alcohol consumption), and physical activity. Physical activity was assessed via self-report questionnaires; for this study, we focused on leisure-time moderate-intensity physical activity hours per week (average hours of moderate exercise such as brisk walking, cycling, etc.), as well as vigorous activity and walking hours for correlation analyses. Sedentary time (daily sitting) was also recorded. Dietary factors were assessed by 24 h dietary recall conducted by trained dietitians in each participant’s home. We extracted total energy intake (kcal per day) and intake of macronutrients (grams per day of carbohydrate, fat, and protein) as well as selected micronutrients (including dietary fiber, calcium, iron, vitamins and others that emerged from preliminary analyses). Anthropometric measurements (body weight, height, and waist circumference) were taken by trained examiners using standardized methods; BMI was calculated as weight (kg)/height (m^2^).

## 4. Statistical Analysis

All analyses accounted for the complex sampling survey designed to produce representative population estimates and correct standard errors. To ensure the national representativeness of the findings and to account for the complex, multi-stage probability sampling design of KNHANES, all statistical analyses were conducted using survey-weighted estimation methods. This approach strictly incorporates sampling weights, stratification (strata), and primary sampling units (clusters) to calculate accurate population-level estimates and unbiased standard errors.

To account for physiological differences and lifestyle patterns, all major statistical analyses were stratified by age and gender. The study subjects were strictly divided into four subgroups based on gender (male and female) and age (younger age group: 20–39 years; middle-aged age group: 40–59 years).

Means with standard errors (SE) were computed for continuous variables and proportions for categorical variables, using weighted survey commands. Differences between groups were tested with chi-square tests as appropriate.

To assess trends over time, we calculated the yearly weighted mean HDL-C from 2014 to 2023 and performed a survey linear regression of HDL-C on survey year (treated as an ordinal variable) to test for a linear trend. We also used one-way complex-sample ANOVA to compare mean HDL-C across the ten survey years. Specifically, for comparing continuous variables across different survey years or demographic groups, we utilized a survey-weighted analysis of variance (ANOVA) employing the design-adjusted Wald F-test. This method is specifically intended for complex survey data, effectively replacing the standard ANOVA, which assumes simple random sampling and would otherwise inappropriately underestimate standard errors. followed by Bonferroni-adjusted pairwise comparisons (post hoc) to identify which years differed significantly from others. Similar trend analyses were conducted for key determinants: mean energy intake, carbohydrate intake, and smoking prevalence by year.

We determined partial correlation coefficients adjusted for age to account for confounding by age distribution. Variables correlated with HDL-C at *p* < 0.05 in age-adjusted analysis were considered for inclusion in multivariable modeling. We then performed a complex-sample multivariable linear regression with HDL-C as the outcome. All statistical tests were two-sided, and *p* < 0.05 was considered statistically significant. Analyses were conducted using R Studio (R version 4.4.1) [18] in accordance with the KNHANES analytical guidelines [19].

## 5. Results

### 5.1. General Characteristics of Study Subjects and HDL-C Levels According to Complex Sampling

In the young male group (aged 20–39 years), significant differences in HDL-C levels were observed according to socioeconomic factors, specifically education level (*p* = 0.024) and individual income (*p* = 0.023). Regarding health behavioral factors, alcohol consumption, smoking, and perceived stress all demonstrated strong associations with HDL-C. Alcohol drinkers exhibited significantly higher HDL-C levels (50.49 mg/dL) compared to non-drinkers (47.41 mg/dL) (*p* < 0.001), whereas current smokers had significantly lower HDL-C levels than non-smokers (48.52 vs. 50.30 mg/dL, *p* < 0.001). Additionally, the group with high perceived stress showed lower HDL-C levels than the low-stress group (*p* = 0.002). Notably, participants taking medication for dyslipidemia presented with lower HDL-C levels compared to non-users (42.75 vs. 49.74 mg/dL, *p* < 0.001), which likely reflects the impact of their underlying metabolic conditions.

In the young female group (aged 20–39 years), a clear positive correlation was observed between socioeconomic status (SES) and HDL-C levels. Higher education levels, individual income, and household income were all associated with significantly higher HDL-C levels (all *p* < 0.001). Regarding employment status, the employed group demonstrated significantly higher HDL-C levels (60.39 mg/dL) than the unemployed group (58.43 mg/dL) (*p* < 0.001). In terms of health behaviors, similar to men, alcohol drinkers had higher HDL-C levels than non-drinkers (*p* < 0.001); however, unlike men, no significant difference was observed according to smoking status (*p* = 0.458). Furthermore, the low-stress group exhibited significantly higher HDL-C levels compared to the high-stress group (*p* = 0.001).

In the middle-aged male group (aged 40–59 years), unlike their younger counterparts, no statistically significant differences were found based on education level (*p* = 0.234) or individual income (*p* = 0.413). However, significant differences were observed according to household income (*p* = 0.049) and employment status (*p* = 0.034). Lifestyle factors showed a pattern similar to the younger group: alcohol drinkers had higher HDL-C than non-drinkers (*p* < 0.001), smokers had lower HDL-C than non-smokers (*p* < 0.001), and the high-stress group exhibited lower HDL-C levels (*p* = 0.004). Meanwhile, in this age group of men, the difference in HDL-C according to dyslipidemia medication use was not statistically significant (*p* = 0.197).

In the middle-aged female group (aged 40–59 years), similar to younger women, socioeconomic factors were shown to have a strong impact on HDL-C levels. Higher education levels, individual income, and household income were consistently associated with significantly higher HDL-C levels (all *p* < 0.001). Among lifestyle factors, alcohol consumption showed a significant difference (*p* < 0.001), whereas smoking (*p* = 0.356) and perceived stress (*p* = 0.402) did not show significant associations with HDL-C levels. Additionally, participants taking dyslipidemia medication had significantly lower HDL-C levels compared to non-users (55.63 vs. 57.75 mg/dL, *p* < 0.001) (Table 1).

### 5.2. Complex Sampling Correlation Analysis Focused on HDL-C

In all groups, waist circumference and body mass index (BMI) showed the strongest and most consistent negative correlations with HDL-C (all groups *p* < 0.001). Notably, these correlations were relatively more pronounced in young adults (aged 20–39 years) than in middle-aged adults (aged 40–59 years) (Male 20–39: waist circumference r = −0.354, BMI r = −0.328; Female 20–39: waist circumference r = −0.331, BMI r = −0.339). This demonstrates a clear inverse relationship, indicating that higher degrees of adiposity are associated with lower HDL-C levels.

The duration of high- and moderate-intensity leisure-time physical activity showed significant positive correlations with HDL-C in most groups, excluding women aged 20–39. Conversely, sedentary time exhibited significant negative correlations across both male groups (Male 20–39: r = −0.041, *p* = 0.006; Male 40–59: r = −0.048, *p* < 0.001), suggesting that longer sitting hours are associated with lower HDL-C levels in men. In women, however, a consistent inverse correlation between sedentary time and HDL-C was not observed.

Among dietary factors, the intake of fat, its subcategories (saturated, monounsaturated, and polyunsaturated fatty acids), and cholesterol showed significant positive correlations with HDL-C across all four groups. This trend was particularly prominent in women (both Female 20–39 and Female 40–59, *p* < 0.001).

Notably, carbohydrate intake exhibited different patterns depending on the age group. In middle-aged adults (40–59 years), both men and women showed significant negative correlations, indicating that higher carbohydrate intake is associated with decreased HDL-C levels (Male: r = −0.046, Female: r = −0.056, both *p* < 0.001). In contrast, young men (20–39 years) showed a weak positive correlation (r = 0.039, *p* = 0.005), while the association was not significant in young women (20–39 years). Regarding other micronutrients, the intakes of water, calcium, and vitamin B2 generally demonstrated significant positive correlations with HDL-C across multiple groups (Table 2).

### 5.3. Factors Affecting HDL Cholesterol

Table 3 presents the results of the complex-sample multiple linear regression analysis conducted to identify the independent determinants affecting HDL-C levels by sex and age group. The explanatory power of the models (R^2^) ranged from 14.1% to 17.6% across the groups.

As a central finding of this study, carbohydrate intake was identified as a robust factor independently lowering HDL-C levels across all four groups. Even after multivariable adjustment, increased carbohydrate intake was significantly associated with decreased HDL-C (Male 20–39: β = −0.131, *p* < 0.001; Female 20–39: β = −0.134, *p* = 0.001; Male 40–59: β = −0.187, *p* < 0.001), and this impact was particularly pronounced in the middle-aged female group (40–59 years) (β = −0.300, *p* < 0.001). Conversely, total energy intake demonstrated an independent positive association with HDL-C in all groups. Among micronutrients, total dietary fiber intake was found to significantly contribute to the elevation of HDL-C in women aged 40–59 (β = 0.064, *p* = 0.001).

Obesity indicators were major factors exerting a consistent negative impact on HDL-C levels. Waist circumference was an independent factor significantly reducing HDL-C in all four groups (all groups *p* < 0.001). Body mass index (BMI) also demonstrated a strong and significant association with reduced HDL-C in three groups, excluding men aged 20–39 (*p* < 0.001). This suggests that, even when controlling all dietary and lifestyle factors, both abdominal and general obesity independently contribute to the reduction in HDL-C.

Among lifestyle factors, alcohol drinking was a common determinant that significantly increased HDL-C across all age and sex groups (all groups *p* < 0.001). In contrast, cigarette smoking acted as a strong risk factor, independently lowering HDL-C across all male groups (both 20–39 and 40–59 years) (*p* < 0.001). In women, no significant effect of smoking was observed; however, a consistent positive association was confirmed where higher educational levels led to significantly increased HDL-C levels (*p* < 0.001) in both young and middle-aged women. Regarding physical activity, moderate-intensity leisure-time physical activity (Moderate PA—Leisure) emerged as a significant factor contributing to HDL-C elevation in men aged 20–39 (*p* < 0.001) and women aged 40–59 (*p* = 0.001) (Table 3).

### 5.4. Trends in Major Factors Affecting HDL-C and Carbohydrate Intake

**Figure 1:**
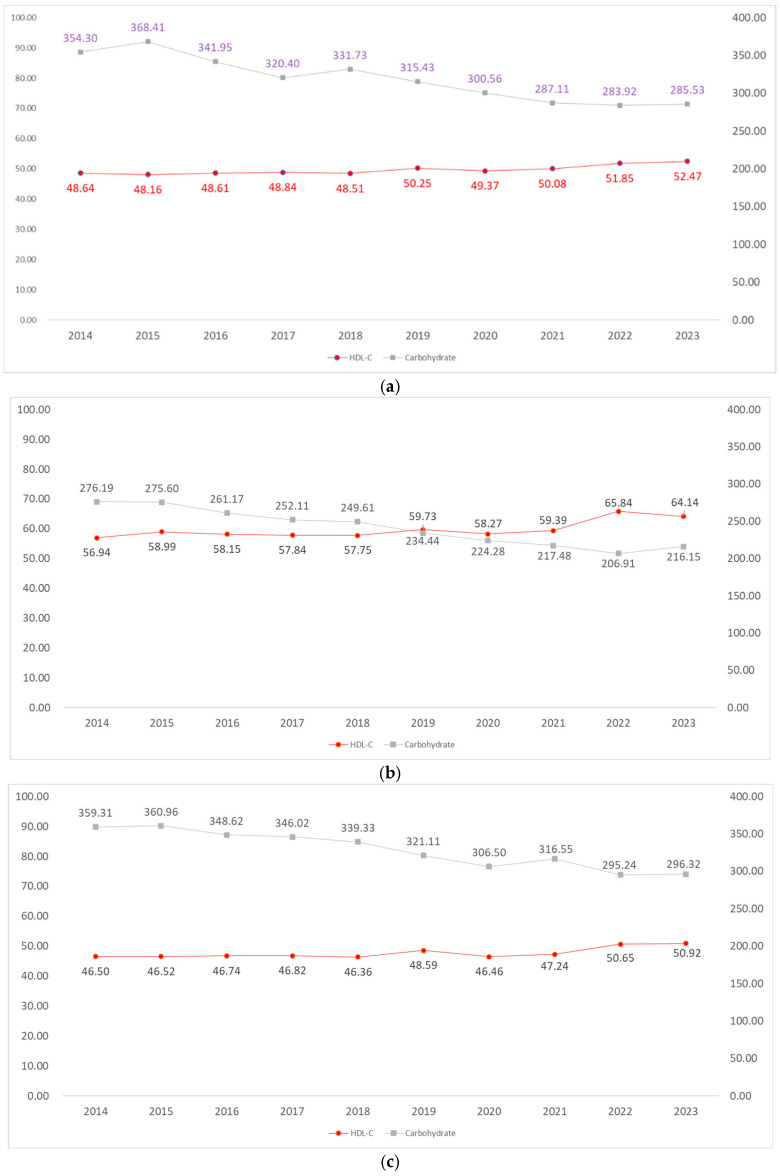

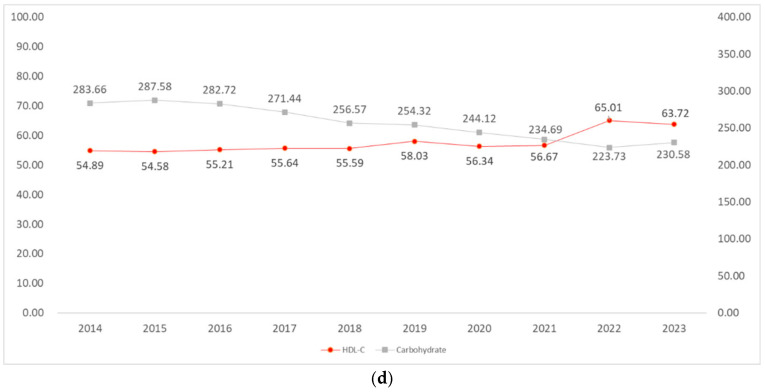
(**a**). Trends in the relationship between HDL-C and Carbohydrate Intake—Male 20–39; Post hoc group: a = 2014, b = 2015, c = 2016, d = 2017, e = 2018, f = 2019, g = 2020, h = 2021, i = 2022, j = 2023; Carbohydrate Intake Trends: Post hoc: Bonferroni = a, b > c, d, e, f > g, h, i, j; HDL-C Trends Post hoc: Bonferroni = a, b, c, d, e < f, g, h, i, j; (**b**). Trends in the relationship between HDL-C and Carbohydrate Intake—Female 20–39; Post hoc group: a = 2014, b = 2015, c = 2016, d = 2017, e = 2018, f = 2019, g = 2020, h = 2021, i = 2022, j = 2023; Carbohydrate Intake Trends: Post hoc: Bonferroni = a, b, c > d, e > f, g, h, i, j; HDL-C Trends Post hoc: Bonferroni = a, b, c, d, e < f, g, h < i, j; (**c**). Trends in the relationship between HDL-C and Carbohydrate Intake—Male 40–59; Post hoc group: a = 2014, b = 2015, c = 2016, d = 2017, e = 2018, f = 2019, g = 2020, h = 2021, i = 2022, j = 2023; Carbohydrate Intake Trends: Post hoc: Bonferroni = a, b, c, d, e > f > g, h, i, j; HDL-C Trends Post hoc: Bonferroni = a, b, c, d, e < f, g, h < i, j; (**d**). Trends in the relationship between HDL-C and Carbohydrate Intake—Female 40–59; Post hoc group: a = 2014, b = 2015, c = 2016, d = 2017, e = 2018, f = 2019, g = 2020, h = 2021, i = 2022, j = 2023; Carbohydrate Intake Trends: Post hoc: Bonferroni = a, b, c, d > e, f > g, h, i, j; HDL-C Trends Post hoc: Bonferroni = a, b, c, d, e < f, g, h < i, j.

Figure 1a–d illustrates the 10-year temporal trends of carbohydrate intake and HDL-C levels from 2014 to 2023 across four subgroups stratified by sex and age (men and women aged 20–39 and 40–59 years). These graphs clearly demonstrate a consistent, macroscopic inverse trajectory between the two indicators across all demographic subgroups.

In all four groups, carbohydrate intake (gray line) depicted a distinct downward curve over the past decade. Statistical post hoc analysis (Bonferroni) revealed that carbohydrate intake was highest during the early years of the study (2014–2016; a, b, c) and exhibited a stepwise declining pattern toward the later period from 2019 onward (f–j), a trend consistently observed regardless of sex and age.

In exact alignment with the declining trajectory of carbohydrate intake, HDL-C levels (red line) displayed a gradual upward trend across all four groups. Post hoc analyses also confirmed that the HDL-C levels in the later half of the study period (2019–2023; f–j) were statistically significantly higher than the average levels in the earlier half (2014–2018; a–e) across all groups. In particular, a more pronounced upward shift was commonly observed starting around 2019 and 2022.

The integrated results of Figure 1 visually and statistically demonstrate that the ‘reduction in carbohydrate intake’ and the ‘elevation of HDL-C’ are synchronized chronologically, forming a perfect cross-over pattern across the entire Korean adult population, rather than being confined to a specific age group (20–30 s vs. 40–50 s) or sex. This strongly corroborates that the cross-sectional association derived from the multivariable regression analysis (Table 3)—identifying carbohydrate intake as an independent risk factor that lowers HDL-C—is in exact alignment with the macroscopic, population-level trend of national dietary transition over the past decade (Figure 1a–d).

## 6. Discussion

### 6.1. Principal Findings and Dietary/Lifestyle Shifts

In this nationwide study of Korean adults, we observed a marked upward trend in HDL cholesterol levels from 2014 to 2023, indicating an overall improvement in population-level metabolic health. Mean HDL-C rose by about 6 mg/dL over the decade, and the prevalence of low HDL-C (a risk factor and diagnostic criterion for metabolic syndrome) decreased dramatically. This favorable trend appears to reflect the net impact of several concurrent public health developments in Korea: most prominently, a significant shift in diet composition and a reduction in smoking. Our analysis suggests that the traditional Korean high-carbohydrate dietary pattern—long implicated in low HDL levels—has attenuated in recent years, with people eating fewer refined carbs and relatively more fats and protein [20]. This nutritional transition is likely a key driver of the improved HDL-C profiles in the population. Meanwhile, sustained tobacco control efforts [19] have lowered smoking rates, removing a source of HDL suppression for many individuals. These positive changes seem to outweigh negative influences such as rising obesity, at least in terms of shaping this specific metabolic biomarker. From a public health perspective, the improvement in HDL-C is encouraging, as low HDL-C has been widespread in Asia [21] and serves as a strong proxy for insulin resistance and elevated cardiometabolic risk. Our findings align with other recent data: a 20-year analysis showed low HDL-C prevalence in Korea nearly halved between 2001 and 2020, especially in women. Interestingly, that study attributed the HDL increase largely to decreased carbohydrate and increased unsaturated fat intake in the Korean diet—precisely the mechanism our data support [21]. Women historically had particularly low HDL in Korea (despite women usually having higher HDL than men globally), likely due to cultural dietary factors; the dramatic HDL gains in Korean women in the past decade have narrowed this gap [22].

The public health implications of these findings are significant. First, they underscore the impact that population-level dietary changes can have on cardiovascular risk factors. Over the past decade, Korean national health campaigns have targeted reduction in sodium and refined carbohydrate intake (including a “Reduction of White Rice Consumption” initiative and promotion of more diverse staples and healthy oils)—although not originally aimed at HDL, these efforts may have yielded the unintended benefit of improving systemic metabolic profiles, as reflected by rising HDL-C levels. It suggests that encouraging a balanced diet with moderate fat intake (particularly unsaturated fats from fish, nuts, and plant oils) in place of excessive carbohydrates can improve lipid profiles in high-carb-consuming populations. This is consistent with clinical trial evidence that low-carbohydrate or Mediterranean-style diets tend to raise HDL-C more than low-fat, high-carb diets [23,24]. For East Asian countries experiencing a nutrition transition, our results provide evidence in support of dietary guidelines that emphasize reducing refined grains and added sugars while incorporating healthy fats. Of course, any dietary strategy must be culturally acceptable and nutritionally adequate [25,26,27]. One concern is that the observed drop in whole-grain intake and certain micronutrients (like thiamine and iron) in Korea could pose other health challenges [28,29]. Policymakers should ensure that while carbohydrate quantity is reduced, the quality of diet remains high (e.g., replacing polished rice with vegetables, legumes, and sources of unsaturated fats rather than with processed meats or sugary foods). Additionally, our multivariable analysis highlights the significant impact of alcohol consumption, iron, and certain vitamins on HDL-C levels, which warrants attention. Consistent with previous studies, alcohol drinking was strongly and positively associated with HDL-C across all subgroups. While alcohol is known to increase the transport rates of apolipoproteins A-I and A-II [30], it also increases HDL-C levels by suppressing cholesteryl ester transfer protein (CETP) activity. Importantly, an increase in HDL-C driven by this CETP suppression is generally not expected to confer anti-atherosclerotic benefits [31]. We caution against initiating alcohol consumption solely for this lipid benefit due to its well-documented adverse health effects [32]. Regarding micronutrients, iron intake showed an inverse relationship with HDL-C, particularly in older groups [33]. This may be explained by the pro-oxidant nature of iron; excess iron accumulation can induce oxidative stress and lipid peroxidation [34], potentially impairing HDL functionality and accelerating its clearance [35]. Conversely, certain vitamins (such as vitamins B1 and B2) exhibited significant associations with HDL-C [36]. B-complex vitamins act as essential coenzymes in cellular energy and lipid metabolism [37]. The protective associations observed may reflect not only the direct role of these vitamins in maintaining metabolic homeostasis but also their role as surrogate markers for a generally high-quality, nutrient-dense diet [38].

### 6.2. The Role of Adiposity and Insulin Resistance

The strong inverse association we found between adiposity and HDL-C reinforces the importance of obesity prevention in cardiometabolic health. The rise in obesity in Korea (though modest compared to Western countries) is an alarming trend that could undermine gains in HDL and other risk factors. Higher BMI and waist circumference each independently predict lower HDL, as well as higher triglycerides and insulin resistance [39]. Therefore, public health policies should intensify focus on healthy weight through interventions like promoting physical activity, improving food environments, and addressing sedentary lifestyles and caloric over-consumption. Notably, our results show physical activity’s direct effect on HDL was quite small after accounting for other factors; however, physical activity is critical for weight control and has many other cardiovascular benefits beyond merely elevating this lipid marker. We interpret this not as physical activity being unimportant, but rather that activity alone (without weight loss or dietary change) has limited capacity to raise HDL by more than a few points. Effective strategies will combine diet, exercise, and weight management to maximize metabolic improvements [40].

The strong inverse association between adiposity indicators (BMI and waist circumference) and HDL-C levels highlights the pivotal role of insulin resistance and glucose tolerance in lipid metabolism. Central obesity, reflected by increased waist circumference, is a primary driver of systemic insulin resistance. When this metabolically compromised state is coupled with a high-carbohydrate diet, it exacerbates postprandial glucose spikes and hyperinsulinemia. From a mechanistic standpoint, insulin resistance decreases the activity of lipoprotein lipase (LPL) and increases hepatic lipase activity, a combination that accelerates the catabolism and renal clearance of HDL particles, thereby lowering circulating HDL-C mass [41,42]. In this pathophysiological context, the importance of physical activity must be emphasized alongside dietary modifications. Although our multivariable model showed a relatively modest direct effect of exercise duration on HDL-C mass, regular physical activity profoundly improves skeletal muscle glucose uptake and systemic insulin sensitivity [43]. Therefore, mitigating insulin resistance through a synergistic combination—reducing refined carbohydrate intake, managing central adiposity, and maintaining regular exercise habits—is essential. This integrated lifestyle approach not only optimizes metabolic biomarkers like HDL-C but also provides comprehensive protection against the broader spectrum of cardiometabolic diseases [44].

### 6.3. Impact of the COVID-19 Pandemic and Clinical Implications

Some intriguing aspects merit further discussion. The surge in mean HDL-C in 2022 was larger than anticipated. While we have attributed it to ongoing trends, one cannot exclude the influence of the COVID-19 pandemic context. It is possible that lifestyle changes during the pandemic, such as reduced dining out, led to healthier home cooking and a reduction in sugary drinks or carb-heavy convenience foods, which in turn improved HDL. Additionally, a proportion of the population may have become more health-conscious in response to COVID-19, potentially affecting lipid levels [45]. Conversely, other reports noted weight gain and worse dietary habits in some groups during lockdowns, which might have temporarily offset HDL gains in 2020 [46]. By 2022, those effects might have normalized, and the underlying positive trend reasserted itself strongly. The large jump in 2022 HDL could also be partly due to random variation or sampling; although KNHANES is carefully designed, the specific sample in 2022 might, by chance, have had slightly more health-aware individuals. We advise interpreting the 2022 value with caution until additional years (2024 onward) confirm whether HDL has truly “reset” at a higher plateau or if it oscillates. Regardless, the consistent upward direction over the decade is clear.

From a global perspective, the Korean experience provides an interesting contrast to Western countries. In the US and Europe, mean HDL-C has been relatively stable or even declining slightly in some cohorts, partly due to obesity and physical inactivity increases, though low-HDL prevalence is generally lower than it was in Asia. Korea’s improvement in HDL-C despite rising obesity highlights the power of dietary composition changes and smoking reduction. Crucially, the population-level rise in HDL observed here should be interpreted primarily as a reflection of broader lifestyle improvements that confer cardiovascular benefits (for example, eating more unsaturated fats and less refined carbs not only raises HDL but also lowers triglycerides and improves insulin sensitivity). Thus, rising HDL can be viewed as a marker of positive health behavior change, rather than a direct goal. We caution that a high HDL-C level per se is not automatically protective—recent Mendelian randomization studies and pharmacologic trials have demonstrated that simply elevating HDL by drug therapy does not necessarily translate into fewer heart attacks [3,47]. The quality and function of HDL particles, and the context of other risk factors, matter. In Korea’s case, the HDL increase has come alongside dropping smoking rates. The net impact on cardiovascular disease outcomes will depend on all these factors. Encouragingly, national data show a decline in ischemic heart disease mortality in Korea in the 2010s, which might be linked in part to these risk factor trends [48].

### 6.4. Limitations

First, the data are observational and cross-sectional in nature (even though we looked at trends across time, each year’s data is a cross-section), so causal inference must be made cautiously.

Second, dietary intake was assessed by a single 24 h recall, which is subject to measurement error and day-to-day variability. While the large sample size should even out random error, some systematic bias might exist. The trend of decreasing carbohydrate intake is supported by multiple years of data and external studies, but the absolute values might be underestimated. However, this methodology is used as a standardized and validated tool within the KNHANES protocol and is therefore useful for national epidemiological surveys.

Third, we did not extensively stratify our analysis by sex or age, as our goal was to examine the overall national trends.

Fourth, as real-world data, various variables potentially related to HDL-C may exist, but due to the nature of secondary data provision, we could not reflect all of them. However, since we utilized publicly available national healthcare big data, we can be confident in the reliability of the measured variables.

Our findings are robust in illustrating the broad shifts in HDL-C and what likely drove them. The public health message is that population-wide lifestyle modifications—even modest ones—can substantially improve lipid profiles. Korea’s case demonstrates that lowering national carbohydrate intake and smoking prevalence, even as BMI rises slightly, was associated with higher HDL-C levels in the populace. For policymakers and health professionals, this emphasizes a multipronged approach: continue to advocate for balanced diets (less sugar and refined grain, more healthy fats and fiber), smoke-free living, and also tackle obesity with urgency. Each of these factors influences different metabolic pathways; together they shape cardiovascular risk.

## 7. Conclusions

In conclusion, HDL cholesterol levels among Korean adults have significantly increased over the last ten years, reflecting a positive development in cardiovascular risk factor status. This improvement correlates with lifestyle changes, notably dietary transition and smoking reduction, amid relatively stable or mildly worsening other factors. While high HDL-C should not be viewed as an end in itself, it serves as an indicator that Korean public health efforts to improve diet and reduce tobacco use are having measurable success. Going forward, sustaining these trends and addressing remaining challenges will be crucial. These findings contribute to a growing body of evidence that favorable changes in population diet and behavior can ameliorate components of metabolic syndrome. Other countries undergoing similar nutrition transitions may draw lessons from the Korean experience—namely, that reducing excessive carbohydrate intake and curbing smoking can lead to meaningful improvements in HDL-C and presumably cardiovascular health. Continuous monitoring of risk factor trends and outcomes will be important to evaluate long-term impacts, especially as Korea and other nations grapple with the dual burden of an aging population and lifestyle-related chronic diseases. Public health strategies that promote a heart-healthy lifestyle at the population level remain a cornerstone of cardiovascular disease prevention in the 21st century.

## Figures and Tables

**Table 1:** General characteristics of study subjects and HDL-C levels according to Complex Sampling.

Variable	Categories	Male 20–39							Female 20–39						
		HDL-C			Weighted		Unweighted		HDL-C			Weighted		Unweighted	
		M	SE	*p*	*n*	%	*n*	%	M	SE	*p*	*n*	%	*n*	%
**Education**	**College**	49.29	0.23	0.024	3,757,476	55.1	2855	56.9	60.32	0.22	<0.001	4,019,276	65.9	4310	67.0
	**High**	50.07	0.26		2,946,571	43.2	2078	41.4	58.56	0.34		1,946,767	31.9	1983	30.8
	**Middle**	51.28	1.52		90,766	1.3	69	1.4	54.27	1.31		102,030	1.7	104	1.6
	**Elementary**	48.16	3.95		18,830	0.3	14	0.3	48.98	2.01		31,059	0.5	37	0.6
**Income**	**High**	50.54	0.35	0.023	1,706,253	25.0	1246	24.8	61.16	0.37	<0.001	1,528,412	25.1	1587	24.7
	**Middle-High**	49.59	0.33		1,685,804	24.7	1265	25.2	59.77	0.37		1,540,444	25.3	1637	25.4
	**Middle-Low**	49.43	0.35		1,746,009	25.6	1272	25.4	59.29	0.37		1,518,515	24.9	1637	25.4
	**Low**	49.05	0.37		1,675,577	24.6	1233	24.6	58.17	0.37		1,511,761	24.8	1573	24.4
**Household Income**	**High**	50.08	0.29	0.227	2,541,396	37.3	1685	33.6	60.92	0.33	<0.001	2,142,811	35.1	2192	34.1
	**Middle-High**	49.55	0.30		2,320,595	34.1	1720	34.3	59.32	0.32		2,096,040	34.4	2259	35.1
	**Middle-Low**	49.08	0.40		1,410,202	20.7	1038	20.7	58.14	0.38		1,430,816	23.5	1561	24.3
	**Low**	49.61	0.64		541,450	7.9	393	7.8	59.26	0.76		429,465	7.0	422	6.6
**Occupation**	**Yes**	49.55	0.20	0.317	4,974,041	73.0	3777	75.3	60.39	60.39	<0.001	3,648,225	59.8	3819	59.4
	**No**	49.94	0.34		1,839,603	27.0	1239	24.7	58.43	58.43		2,450,976	40.2	2615	40.6
**Alcohol Drinking**	**Yes**	50.49	2.21	<0.001	4,967,888	72.9	3685	73.5	61.20	61.20	<0.001	3,641,063	59.7	3785	58.8
	**No**	47.41	0.30		1,845,755	27.1	1331	26.5	57.24	57.24		2,458,069	40.3	2649	41.2
**Cigarette Smoking**	**Yes**	48.52	0.29	<0.001	2,460,524	36.1	1834	36.6	59.10	59.10	0.458	502,032	8.2	485	7.5
	**No**	50.30	0.22		4,353,120	63.9	3182	63.4	59.65	59.65		5,597,100	91.8	5949	92.5
**Stress**	**High**	48.84	0.31	0.002	2,161,706	31.7	1617	32.2	58.84	58.84	0.001	2,371,250	38.9	2462	38.3
	**Low**	50.03	0.21		4,651,937	68.3	3399	67.8	60.09	60.09		3,727,882	61.1	3972	61.7
**Medication for dyslipidemia**	**Yes**	42.75	1.07	<0.001	77,833	1.1	60	1.2	53.10	2.79	0.019	26,466	0.4	37	0.6
	**No**	49.74	0.18		6,735,810	98.9	4956	98.8	59.63	0.19		6,069,666	99.5	6397	99.4
**Total**		49.44	0.26		6,813,643	100.0	5016	100.0	59.47	59.47		6,099,132	100.0	6434	100.0
**Variable**	**Categories**	**Male 40–59**							**Female 40–59**						
		**HDL-C**			**Weighted**		**Unweighted**		**HDL-C**			**Weighted**		**Unweighted**	
		**M**	**SE**	***p***	***n***	**%**	***n***	**%**	**M**	**SE**	***p***	***n***	**%**	***n***	**%**
**Education**	**College**	47.73	0.21	0.234	4,014,670	53.1	3495	52.2	59.37	0.28	<0.001	3,120,571	41.5	4110	41.3
	**High**	47.65	0.27		2,703,223	35.8	2374	35.5	56.96	0.24		3,330,634	44.3	4309	43.3
	**Middle**	47.86	0.58		547,370	7.2	526	7.9	54.35	0.56		640,717	8.5	907	9.1
	**Elementary**	46.23	0.72		294,131	3.9	298	4.5	53.22	0.58		425,144	5.7	632	6.3
**Income**	**High**	47.97	0.30	0.413	1,897,101	25.1	1676	25.0	58.84	0.33	<0.001	1,922,028	25.6	2526	25.4
	**Middle-High**	47.82	0.30		1,956,111	25.9	1704	25.5	57.66	0.33		1,924,124	25.6	2520	25.3
	**Middle-Low**	47.47	0.32		1,904,770	25.2	1663	24.8	56.85	0.32		1,914,951	25.5	2504	25.1
	**Low**	47.33	0.31		1,801,412	23.8	1650	24.7	56.68	0.32		1,755,963	23.4	2408	24.2
**Household** **Income**	**High**	47.99	0.24	0.049	3,127,300	41.4	2750	41.1	58.49	0.28	<0.001	2,939,245	39.1	3837	38.5
	**Middle-High**	47.54	0.27		2,410,700	31.9	2087	31.2	57.38	0.29		2,358,091	31.4	3074	30.9
	**Middle-Low**	47.63	0.34		1,468,559	19.4	1329	19.9	56.79	0.34		1,644,293	21.9	2227	22.4
	**Low**	46.26	0.59		552,835	7.3	527	7.9	55.38	0.49		575,437	7.7	820	8.2
**Occupation**	**Yes**	47.76	0.16	0.034	6,879,507	91.0	6076	90.8	57.68	0.20	0.235	4,666,470	62.1	6309	63.4
	**No**	46.59	0.53		679,887	9.0	617	9.2	57.28	0.27		2,850,596	37.9	3649	36.6
**Alcohol Drinking**	**Yes**	48.63	0.19	<0.001	5,520,322	73.0	4885	73.0	59.87	0.25	<0.001	3,481,787	46.3	4540	45.6
	**No**	45.00	0.26		2,039,072	27.0	1808	27.0	55.51	0.20		4,035,279	53.7	5418	54.4
**Cigarette Smoking**	**Yes**	46.64	46.64	<0.001	2,888,498	38.2	2593	38.7	56.91	0.82	0.356	345,295	4.6	453	4.5
	**No**	48.28	48.28		4,670,896	61.8	4100	61.3	57.56	0.17		7,171,771	95.4	9505	95.5
**Stress**	**High**	46.92	46.92	0.004	2,058,797	27.2	1806	27.0	57.29	0.33	0.402	1,954,281	26.0	2653	26.6
	**Low**	47.93	47.93		5,500,597	72.8	4887	73.0	57.61	0.19		5,562,785	74.0	7305	73.4
**Medication for** **dyslipidemia**	**Yes**	48.18	0.44	0.197	905,415	12.0	835	12.5	55.63	0.50	<0.001	783,648	10.4	1070	10.7
	**No**	47.58	0.17		6,653,979	88.0	5858	87.5	57.75	0.18		6,733,418	89.6	8887	89.2
**Total**		47.43	47.43		7,559,394	100.0	6693	100.0	57.45	0.26		7,517,066	100.0	9958	100.0

**Table 2:** Complex Sampling Correlation Analysis Focused on HDL-C.

Variables	Male 20–39				Female 20–39			
	M	SE	r	*p*	M	SE	r	*p*
HDL-C (mg/dL)	49.66	0.17	1	1	59.6	0.19	1	1
High Physical Activity—Work (minutes)	3.92	0.38	0.009	0.493	0.98	0.18	0.007	0.605
Moderate Physical Activity—Work (minutes)	16.11	0.86	0.016	0.275	11.73	0.72	0.010	0.464
Location Move Physical Activity (minutes)	25.64	0.5	0.028	0.052	26.39	0.46	−0.018	0.000
High Physical Activity—Leisure (minutes)	15.12	0.55	0.077	<0.001	5.38	0.24	0.045	0.184
Moderate Physical Activity—Leisure (minutes)	19.92	0.54	0.073	<0.001	13.84	0.37	0.046	0.003
Walk (minutes)	46.63	0.78	0.007	0.618	48.79	0.72	−0.003	0.839
Sedentary (minutes)	551.91	3.51	−0.041	0.006	544.94	3.16	0.033	0.023
Waist circumference (cm)	86.16	0.17	−0.354	<0.001	74.62	0.15	−0.331	<0.001
Body Mass Index (kg/m^2^)	24.94	0.06	−0.328	<0.001	22.32	0.06	−0.339	<0.001
Energy intake (kcal)	2462.53	17.2	0.030	0.039	1754.87	10.36	0.047	0.001
Water intake (g)	1208.28	11.48	−0.041	0.008	967.77	7.77	0.073	<0.001
Carbohydrate (g)	95.53	0.8	0.039	0.005	65.74	0.49	−0.020	0.152
Protein intake (g)	70.26	0.73	0.021	0.152	50.78	0.47	0.047	0.001
Fat intake (g)	22.37	0.25	0.035	0.023	16.79	0.18	0.075	<0.001
Saturated fatty acids intake (g)	23.53	0.29	0.037	0.019	16.39	0.17	0.069	<0.001
Monounsaturated fatty acids intake (g)	16.87	0.19	0.040	0.011	12.12	0.13	0.066	<0.001
Polyunsaturated fatty acids intake (g)	2.11	0.03	0.024	0.092	1.61	0.02	0.080	<0.001
*n*3 fatty acid (g)	14.74	0.17	−0.002	0.878	10.49	0.11	0.055	<0.001
*n*6 fatty acid (g)	357.61	4.24	0.027	0.060	267.33	2.93	0.079	<0.001
Cholesterol intake (mg)	318.86	2.17	0.024	0.109	241.83	1.46	0.049	<0.001
Total Dietary Fiber intake (g)	23.85	0.21	−0.015	0.320	18.64	0.14	0.006	0.647
Calcium intake (mg)	541.4	4.84	0.005	0.752	450.6	3.82	0.065	<0.001
Phosphorus intake (mg)	1278.16	9.03	0.009	0.570	943.78	6.01	0.042	0.004
Sodium intake (mg)	4252.81	38.41	−0.011	0.423	2964.06	24.69	0.007	0.614
Potassium intake (mg)	3015.95	22.77	−0.008	0.597	2351.85	15.89	0.018	0.216
Iron intake (mg)	13.84	0.16	−0.015	0.342	10.35	0.1	−0.004	0.754
Carotene intake (μg)	2853.99	59.49	0.018	0.564	2329.45	60.74	0.002	0.863
Retinol intake (mg)	213.4	8.54	0.015	0.108	174.91	5.04	0.045	0.010
Vitamin B1 (mg)	1.76	0.02	−0.019	0.196	1.22	0.01	−0.035	0.027
Vitamin B2 (mg)	1.99	0.02	0.028	0.062	1.48	0.01	0.066	<0.001
Niacin (mg)	17.98	0.18	0.021	0.171	12.59	0.11	0.019	0.243
Vitamin C (mg)	69.88	1.87	0.007	0.671	63.16	1.15	0.021	0.151
**Variables**	**Male 40–59**				**Female 40–59**			
	**M**	**SE**	**r**	***p***	**M**	**SE**	**r**	***p***
HDL-C (mg/dL)	47.65	0.16	1	1	57.53	0.17	1	1
High Physical Activity—Work (minutes)	3.24	0.38	0.008	0.419	0.67	0.11	0.034	0.055
Moderate Physical Activity—Work (minutes)	11.66	0.63	0.024	0.090	6.82	0.46	0.022	0.025
Location Move Physical Activity (minutes)	20.35	0.44	0.005	0.684	23.71	0.36	−0.011	0.327
High Physical Activity—Leisure (minutes)	9.59	0.4	0.058	<0.001	4.07	0.2	0.062	<0.001
Moderate Physical Activity—Leisure (minutes)	17.94	0.48	0.040	0.002	13.33	0.35	0.083	<0.001
Walk (minutes)	48.6	0.8	0.038	0.010	51.27	0.6	0.009	0.463
Sedentary (minutes)	500.71	3.33	−0.048	<0.001	460.86	2.57	0.010	0.383
Waist circumference (cm)	87.82	0.12	−0.276	<0.001	78.99	0.12	−0.279	<0.001
Body Mass Index (kg/m^2^)	24.93	0.04	−0.267	<0.001	23.45	0.04	−0.279	<0.001
Energy intake (kcal)	2362.67	13.36	0.040	0.004	1670.29	7.57	0.020	0.071
Water intake (g)	1242.39	10.11	0.044	0.002	1040.55	7.2	0.055	<0.001
Carbohydrate (g)	85.54	0.62	−0.046	<0.001	61.39	0.34	−0.056	<0.001
Protein intake (g)	55.19	0.52	0.022	0.063	40.78	0.3	0.043	<0.001
Fat intake (g)	17.18	0.18	0.033	0.013	12.55	0.11	0.090	<0.001
Saturated fatty acids intake (g)	17.99	0.2	0.029	0.036	13	0.11	0.085	<0.001
Monounsaturated fatty acids intake (g)	14.02	0.14	0.034	0.013	10.76	0.09	0.090	<0.001
Polyunsaturated fatty acids intake (g)	2.12	0.03	0.036	0.006	1.74	0.02	0.073	<0.001
*n*3 fatty acid (g)	11.89	0.12	0.033	0.012	9.01	0.08	0.024	0.124
*n*6 fatty acid (g)	305.17	3.58	0.034	0.012	227	2.23	0.075	<0.001
Cholesterol intake (mg)	328.85	1.79	0.046	<0.001	256.79	1.27	0.080	<0.001
Total Dietary Fiber intake (g)	28.39	0.19	0.006	0.656	24.82	0.17	−0.005	0.656
Calcium intake (mg)	582.18	4.45	0.044	0.002	482.47	3.36	0.041	<0.001
Phosphorus intake (mg)	1258.37	7.68	0.024	0.065	967.39	4.97	0.041	<0.001
Sodium intake (mg)	4356.41	38.98	0.010	0.300	2978.36	20.71	−0.020	0.067
Potassium intake (mg)	3293.13	20.48	0.011	0.447	2759.29	18.38	−0.007	0.553
Iron intake (mg)	14.24	0.13	−0.008	0.542	11.1	0.08	−0.043	<0.001
Carotene intake (μg)	3393.71	46.22	0.007	0.640	3046.38	38.41	−0.011	0.317
Retinol intake (mg)	157.36	5.01	0.029	0.155	136.83	3.05	0.048	<0.001
Vitamin B1 (mg)	1.66	0.01	−0.006	0.663	1.23	0.01	−0.044	<0.001
Vitamin B2 (mg)	1.84	0.01	0.040	0.005	1.43	0.01	0.066	<0.001
Niacin (mg)	16.32	0.14	−0.004	0.785	12.16	0.08	0.002	0.884
Vitamin C (mg)	78.69	1.69	−0.013	0.287	79.65	1.31	0.005	0.686

**Table 3:** Factors Affecting HDL Cholesterol.

Variable	Male 20–39			Female 20–39		
	B	β	*p*	B	β	*p*
(Intercept)	82.578		<0.001	80.004		<0.001
Education	−0.463	−0.022	0.139	1.184	0.049	<0.001
Income	0.110	0.004	0.687	1.304	0.043	0.173
Household Income	−0.043	−0.001	0.769	−2.011	−0.071	0.054
Occupation	0.002	0.000	0.996	0.833	0.031	0.020
Alcohol Drinking	2.945	0.115	<0.001	2.959	0.110	<0.001
Cigarette Smoking	−2.038	−0.086	<0.001	−0.021	0.000	0.974
Stress	−0.208	−0.008	0.547	−0.725	−0.027	0.032
Medication for dyslipidemia	−3.177	−0.030	0.005	0.185	0.001	0.941
High Physical Activity Hour—Work	−0.001	−0.001	0.914	0.010	0.009	0.515
Moderate Physical Activity Hour—Work	0.004	0.022	0.119	0.005	0.020	0.123
Location Move Physical Activity Hour	0.000	0.001	0.971	0.001	0.002	0.889
High Physical Activity Hour—Leisure	0.012	0.036	0.029	0.021	0.027	0.050
Moderate Physical Activity Hour—Leisure	0.020	0.056	<0.001	0.009	0.018	0.187
Walk Hour	0.001	0.002	0.867	0.001	0.005	0.727
Sedentary Hour	−0.002	−0.032	0.029	0.000	0.008	0.569
Waist circumference	−0.352	−0.328	<0.001	−0.142	−0.106	0.001
Body Mass Index	−0.067	−0.024	0.541	−0.757	−0.230	<0.001
Energy intake	0.002	0.153	0.002	0.002	0.141	0.019
Water intake	0.001	0.051	0.011	0.001	0.055	0.008
Carbohydrate	−0.011	−0.131	<0.001	−0.017	−0.134	0.001
Protein intake	0.009	0.042	0.332	0.020	0.051	0.195
Fat intake	−0.158	−0.657	<0.001	−0.078	−0.198	0.236
Saturated fatty acids intake	0.154	0.220	0.004	0.066	0.062	0.416
Monounsaturated fatty acids intake	0.191	0.305	<0.001	0.087	0.079	0.272
Polyunsaturated fatty acids intake	0.842	0.914	0.083	1.466	1.017	0.071
*n*3 fatty acid	−0.922	−0.150	0.069	−1.492	−0.177	0.068
*n*6 fatty acid	−0.654	−0.628	0.174	−1.317	−0.797	0.104
Cholesterol intake	0.000	0.005	0.828	0.000	0.004	0.850
Total Dietary Fiber intake	0.035	0.040	0.092	0.042	0.032	0.135
Calcium intake	0.000	0.010	0.686	0.003	0.059	0.007
Phosphorus intake	−0.001	−0.029	0.570	−0.002	−0.059	0.212
Sodium intake	0.000	0.003	0.865	0.000	−0.004	0.807
Potassium intake	−0.001	−0.075	0.026	0.000	−0.014	0.644
Iron intake	−0.025	−0.022	0.294	−0.049	−0.026	0.148
Carotene intake	0.000	0.034	0.300	0.000	0.012	0.525
Retinol intake	0.000	0.011	0.209	0.001	0.018	0.310
Vitamin B1	−0.674	−0.066	0.003	−1.374	−0.083	<0.001
Vitamin B2	0.172	0.017	0.534	0.427	0.027	0.234
Niacin	0.003	0.003	0.914	−0.061	−0.035	0.178
Vitamin C	0.001	0.008	0.632	0.005	0.029	0.042
R^2^ Score	R^2^ = 0.176			R^2^ = 0.167		
**Variable**	**Male 40–59**			**Female 40–59**		
	**B**	**β**	***p***	**B**	**β**	***p***
(Intercept)	79.272		<0.001	81.676		<0.001
Education	0.138	0.009	0.478	0.799	0.047	<0.001
Income	−0.465	−0.017	0.646	−0.691	−0.022	0.422
Household Income	0.461	0.017	0.625	0.849	0.028	0.302
Occupation	0.595	0.015	0.317	0.211	0.007	0.507
Alcohol Drinking	3.494	0.133	<0.001	3.806	0.136	<0.001
Cigarette Smoking	−2.330	−0.097	<0.001	−0.944	−0.014	0.238
Stress	−0.446	−0.017	0.173	0.035	0.001	0.919
Medication for dyslipidemia	1.936	0.054	<0.001	0.542	0.012	0.283
High Physical Activity Hour—Work	0.002	0.005	0.634	0.047	0.033	0.058
Moderate Physical Activity Hour—Work	0.008	0.030	0.023	0.003	0.008	0.429
Location Move Physical Activity Hour	−0.007	−0.020	0.115	−0.002	−0.004	0.682
High Physical Activity Hour—Leisure	0.015	0.037	0.005	0.021	0.024	0.064
Moderate Physical Activity Hour—Leisure	0.004	0.012	0.359	0.021	0.040	0.001
Walk Hour	0.005	0.025	0.086	0.001	0.005	0.669
Sedentary Hour	−0.001	−0.024	0.068	0.000	−0.005	0.629
Waist circumference	−0.268	−0.199	<0.001	−0.205	−0.136	<0.001
Body Mass Index	−0.425	−0.117	<0.001	−0.546	−0.138	<0.001
Energy intake	0.002	0.199	<0.001	0.007	0.350	<0.001
Water intake	0.000	0.025	0.190	0.001	0.044	0.009
Carbohydrate	−0.017	−0.187	<0.001	−0.038	−0.300	<0.001
Protein intake	0.004	0.014	0.763	−0.012	−0.025	0.430
Fat intake	−0.081	−0.260	0.066	−0.091	−0.172	0.187
Saturated fatty acids intake	0.076	0.084	0.202	0.032	0.021	0.696
Monounsaturated fatty acids intake	0.063	0.076	0.313	0.078	0.053	0.355
Polyunsaturated fatty acids intake	0.279	0.246	0.539	0.280	0.159	0.059
*n*3 fatty acid	−0.116	−0.025	0.798	−0.279	−0.040	0.001
*n*6 fatty acid	−0.181	−0.134	0.687	−0.167	−0.082	0.244
Cholesterol intake	0.001	0.020	0.345	0.001	0.018	0.292
Total Dietary Fiber intake	0.034	0.041	0.054	0.066	0.064	0.001
Calcium intake	0.002	0.051	0.018	0.001	0.031	0.099
Phosphorus intake	−0.001	−0.056	0.292	0.001	0.023	0.554
Sodium intake	0.000	0.001	0.955	0.000	−0.033	0.011
Potassium intake	0.000	0.011	0.684	0.000	−0.050	0.051
Iron intake	−0.048	−0.039	0.039	−0.121	−0.062	<0.001
Carotene intake	0.000	−0.007	0.651	0.000	−0.001	0.909
Retinol intake	0.000	0.002	0.908	0.001	0.013	0.294
Vitamin B1	−0.176	−0.014	0.430	−0.764	−0.041	0.011
Vitamin B2	0.434	0.037	0.113	0.274	0.015	0.473
Niacin	−0.082	−0.075	0.006	−0.128	−0.061	0.002
Vitamin C	−0.001	−0.007	0.567	0.003	0.023	0.069
R^2^ Score	R^2^ = 0.141			R^2^ = 0.142		

## Data Availability

Publicly Accessible Repository. The original data presented in the study are openly available in the Korea National Health and Nutrition Examination Survey (KNHANES), Korea Disease Control and Prevention at https://knhanes.kdca.go.kr/knhanes/eng/main.do (accessed on 5 December 2025) [13].
